# Feeding-induced changes in allatostatin-A and short neuropeptide F in the antennal lobes affect odor-mediated host seeking in the yellow fever mosquito, *Aedes aegypti*

**DOI:** 10.1371/journal.pone.0188243

**Published:** 2017-11-22

**Authors:** Peter Christ, Anna Reifenrath, Jörg Kahnt, Frank Hauser, Sharon Rose Hill, Joachim Schachtner, Rickard Ignell

**Affiliations:** 1 Unit of Chemical Ecology, Department of Plant Protection Biology, Swedish University of Agricultural Sciences, Alnarp, Sweden; 2 Neurobiology/Ethology, Department of Biology, Philipps University Marburg, Marburg, Germany; 3 Max Planck Institute for Terrestrial Microbiology, Marburg, Germany; 4 Center for Functional and Comparative Insect Genomics, Department of Biology, University of Copenhagen, Copenhagen, Denmark; Universidade Federal do Rio de Janeiro, BRAZIL

## Abstract

*Aedes aegypti* is a model species in which the endogenous regulation of odor-mediated host seeking behavior has received some attention. Sugar feeding and host seeking in female *A*. *aegypti* are transiently inhibited following a blood meal. This inhibition is partially mediated by short neuropeptide F (sNPF). The paired antennal lobes (ALs), as the first processing centers for olfactory information, has been shown to play a significant role in the neuropeptidergic regulation of odor-mediated behaviors in insects. The expression of sNPF, along with other peptides in the ALs of *A*. *aegypti*, indicate parallel neuromodulatory systems that may affect olfactory processing. To identify neuropeptides involved in regulating the odor-mediated host seeking behavior in *A*. *aegypti*, we use a semi-quantitative neuropeptidomic analysis of single ALs to analyze changes in the levels of five individual neuropeptides in response to different feeding regimes. Our results show that the level of sNPF-2, allatostatin-A-5 (AstA-5) and neuropeptide-like precursor-1-5 (NPLP-1-5), but not of tachykinin-related-peptides and SIFamide (SIFa), in the AL of female mosquitoes, changes 24 h and 48 h post-blood meal, and are dependent on prior access to sugar. To assess the role of these neuropeptides in modulating host seeking behavior, when systemically injected individually, sNPF-2 and AstA-5 significantly reduced host seeking behavior. However, only the injection of the binary mixture of the two neuropeptides lead to a host seeking inhibition similar to that observed in blood fed females. We conclude that modulation of the odor mediated host seeking behavior of *A*. *aegypti* is likely regulated by a dual neuropeptidergic pathway acting in concert in the ALs.

## Introduction

*Aedes aegypti* is the primary vector of dengue, zika, chikungunya, and yellow fever that affect millions of people annually [1]. These diseases are transmitted by female mosquitoes during blood feeding, which are located during a process known as host seeking, a behavior predominantly driven by olfactory cues [2]. Blood feeding and the ensuing behaviors in female *A*. *aegypti* are regulated by endogenous factors and are dependent on the physiological state of the animal [3,4]. Previous studies have indicated that neuropeptides might be among those endogenous factors [5,6]. The purpose of this study is to identify changes in the expression levels of selected neuropeptides in the primary olfactory center, the antennal lobe (AL), and determine their effect on host seeking in *A*. *aegypti*. The selected neuropeptides were chosen based on previous studies indicating a role of these in affecting feeding-related behaviors in other insects, as well as their amenability to be assayed with isotope-labelled peptides [7–13]. As the first processing center for olfactory information, the paired antennal lobes (ALs) play a significant role in the neuropeptidergic regulation of odor-mediated behaviors, as shown in *Drosophila melanogaster* [14,15].

Blood feeding in *A*. *aegypti* is required for complete egg development, and leads to significant changes in behavior, such as reduced flight activity [16] and a decreased response to host cues [4,17–19]. This induced olfactory unresponsiveness correlates with a reduced physiological response of the olfactory sensory neurons (OSNs) tuned to host cues [20]. Following the completion of egg development, approximately 72 h post-blood meal (pbm), females display pre-oviposition behavior. At this time, both flight activity [16] and the physiological response of the OSNs tuned to egg-laying site cues increase [3,21]. Throughout the reproductive cycle, female mosquitoes require periodic sugar meals to ensure survival and reproductive fitness [22–24]. During blood meal-induced ovarian development, mosquitoes usually do not feed on sugar, while gravid mosquitoes commonly replenish their energy resources before oviposition [23–25]. In sugar-starved mosquitoes, the responsiveness to host cues is fully restored by between 48 h and 72 h pbm, indicating the importance of an additional meal [24]. After egg-laying, the reproductive cycle is complete, and host seeking behavior is re-established within 24 h [3,19].

While the effects of blood feeding on the peripheral olfactory system in mosquitoes have been studied in some detail [20,21,26–29], there are as of yet no studies investigating the effects of blood feeding on the ALs. Research in *D*. *melanogaster* has demonstrated that olfactory behaviors can be modulated by peptidergic neurons innervating the ALs [7,10,12,14,30]. The ALs of both *A*. *aegypti* and *D*. *melanogaster* are innervated by complex modulatory circuits comprised, in part, by peptidergic local interneurons (LNs) and centrifugal neurons [31,32]. In *A*. *aegypti* alone, 28 mature neuropeptides, arising from 10 neuropeptide genes, are expressed in the ALs [32]. Among these, short neuropeptide F (sNPF) has been linked directly to feeding behavior in *A*. *aegypti* and *D*. *melanogaster* [6,7,33]. In addition, tachykinin-related peptides (TKRP) [7,12], and allatostatin-A (AstA) [8,11] have been linked directly to feeding behavior in *D*. *melanogaster*. In *D*. *melanogaster*, sNPF and TKRP neurons interact with other AL neurons, both pre- and post-synaptically [7,12]. While there is only limited information available about other neuropeptides expression profiles with respect to their involvement in odor-related blood feeding behavior, a mass spectrometric study in *Rhodnius prolixus* indicate the neuropeptide-like precursor-1 (NPLP-1) in regulating this behavior [9].

In our study, we use semi-quantitative matrix assisted laser desorption ionization-time of flight (MALDI-TOF) mass spectrometry to identify changes in the level of selected neuropeptides in the ALs of female *A*. *aegypti* throughout the first reproductive cycle, in response to different feeding regimes. A significant and transient change in the levels of the neuropeptide isoforms sNPF-2, AstA-5, and NPLP-1-5 was found following a blood meal. Systemic injection of sNPF-2 and AstA-5, individually partially inhibited, and together fully inhibited, host seeking in non-blood-fed female *A*. *aegypti*.

## Materials and methods

### Animal rearing

*Aedes aegypti* (Rockefeller strain) were kept at 27 ± 1°C, 70% relative humidity, and at a 12 h:12 h light: dark cycle. Larvae were reared in plastic trays (30 cm × 15 cm × 5 cm) filled with distilled water, and fed Tetramin fish flakes (Tetrawerke, Melle, Germany). Pupae were transferred daily into rearing cages (30 cm × 30 cm × 30 cm Bugdorm; Megaview Science, Taiwan) or a plastic bucket (10 L; MALDI-TOF experiments). Adults were given access to a 4% sugar solution for 1 h per day, 2.5 h after light onset. Six days post-eclosion, males were removed from the cages and the females split into two groups. One group (blood fed; bf) was given access to blood, warmed to 37°C, for 1 h, 1.5 h after light onset. For the MALDI-TOF analysis, stirred pig blood (Metzgerei Binzer, Germany) was delivered in a glass vial covered with parafilm, while for the behavioral experiments sheep blood (Håtuna Lab, Sweden) was provided via a membrane feeding system (Hemotek, Discovery Workshops, Accrington, UK). After blood feeding, females were given access to sugar, as described above. Animals that did not feed to repletion on blood were discarded from the cohort. The control group received sugar (non-blood fed; nbf). Female mosquitoes were used 1 h, 24 h, 48 h, and 72 h pbm, as well as 24 h post-egg laying (ca. 120 h pbm). Starting from 96 h pbm, females were provided with an egg-laying substrate consisting of a filter paper in water from the larval trays in a 100 mL polypropylene cup (Qingdao Ori-Color Industry and Commerce Co., Ltd., China). Egg laying status was determined by dissection. On the day of analysis for each experiment, 1 mg mL^-1^ xylene cyanol FF (Sigma-Aldrich, Chemie GmbH, Steinheim, Germany) was added to the 4% sugar solution. Presence of xylene cyanol FF in the gut indicated sugar feeding and was assessed by dissection, and visually confirmed following each experiment. Dissections and behavioral assays were carried out between ZT 5.5 and ZT 10.5, to cover the second host-seeking activity period [16].

### Wind tunnel bioassay

The behavioral response to host cues by nbf and bf *A*. *aegypti* was analyzed in a glass wind tunnel, illuminated from above at 500 lx, as described by Majeed et al. [34]. Charcoal-filtered, humidified air (25 ± 2°C, 65 ± 2% RH) flowed through the wind tunnel at 30 cm s^-1^. Single individuals were kept in plastic release cages for 2 h prior to the experiments. The release cages were then placed at the downwind end of the wind tunnel, and the mosquitoes allowed to acclimatize to the airflow for 60 s before the door of the release cage was opened. Human odors were provided by holding the hand of the experimenter in the airflow at the upwind end of the wind tunnel, behind a capture cage. Attraction was scored if the mosquito made source contact, i.e., landed in the capture chamber within 180 s of release. Following the experiment, mosquitoes were analyzed to determine the presence of xylene cyanol FF in the gut, as described above. The number of females in each feeding state was compared by Fisher's exact test using Graph Pad Prism 6 (GraphPad Software, La Jolla, USA, RRID: SCR_002798). The behavioral responses of nbf and bf mosquitoes were analyzed using nominal logistic regression. Data were statistically analyzed using SAS 9.4 (SAS Institute Inc, Cary, USA, RRID: SCR_008567) and plotted using Graph Pad Prism 6.

### Analysis of sugar feeding behavior

The effect of blood feeding on sugar consumption was determined as the percentage of mosquitoes taking a sugar meal at 1 h, 24 h, 48 h, and 72 h pbm, as well as 24 h post-egg laying (120 h pbm). For each time point, three biological replicates of 20–50 mosquitoes were used. Sugar feeding behavior of blood fed and non-blood fed animals were plotted and compared by a Fisher's exact test using Graph Pad Prism 6.

### Sample preparation

Non-blood fed and bf *A*. *aegypti* from the same cohort were cold anesthetized, and their brains removed from the head capsule into a droplet of ice-cold ammonium chloride (Merck, Darmstadt, Germany). The individual ALs were detached using sharp forceps and transferred to a stainless steel MALDI-TOF plate and dried, as described by Schachtner et al. and Siju et al. [21, 35]. Internal standards [36], consisting of ^13^C-, ^15^N isotope labeled TKRP-1, sNPF-2, AstA-5, NPLP-1-5 and SIFamide (SIFa; PANATecs GmbH, Tübingen, Germany; Table 1), were kept as stocks (10^−2^ M in 30% acetonitrile; Roth, Karlsruhe, Germany) at 4°C. For each experiment, the five isotope labeled peptides were mixed and diluted to 10^−8^ M. A saturated α-cyano-4-hydroxycinnamic acid matrix was prepared in methanol:ethanol:H_2_O:trifluoroacetic acid (30:30:39:1) and mixed with an equal amount of the diluted peptides (0.1 μL) and spotted onto the dried ALs. For each time point, 3 to 4 individual experiments were conducted using a fresh dilution of the peptides.

**Table 1:** Sequence, molecular weight and isotope labelling of investigated neuropeptides.

Neuropeptide	Sequence	*Mean of the monoisotopic mass*			Isotope labelled neuropeptide	*Mean of the monoisotopic mass*		
		Calculated	Measured	Deviation		Calculated	Measured	Deviation
TKRP-1	APSGFLGLR	916.53	916.52	0.0417	APSGFLGL*R	923.53	923.53	0.0418
sNPF-2	APQLRLRF	999.62	999.60	0.0455	APQLRL*RF	1006.62	1006.62	0.0466
AstA-5	LPNRYNFGL	1092.58	1092.58	0.0560	LPNRYNFGL*	1099.58	1099.59	0.0505
NPLP-1-5	NIASLARKYELP	1373.79	1373.77	0.0593	NIA*SLARKYELP	1377.79	1377.77	0.0597
SIFa	GYRKPPFNGSIF	1381.74	1381.72	0.0638	G*YRKPP*FNGSIF	1390.74	1390.73	0.0632

* indicates the isotope labelled amino acid residue

### MALDI-TOF mass spectrometry

Samples were analyzed on a 4800 MALDI TOF/TOF plus analyzer (Applied Biosystems/MDS SCIEX, Foster City, USA), in reflection mode within 24 h of dissection. Each spectrum represents the mean of 3000 laser shots, randomly distributed across the entire sample over a range of mass to valence ratio (m/z) of 900–1500. Mass calibration was performed using standard no. 206195 from Bruker Daltonics (Bremen, Germany; Angiotensin III: 931.515, Angiotensin II: 1046.542, Angiotensin I: 1296.685, ACTH (18–39): 2465.199).

**Fig 1:**
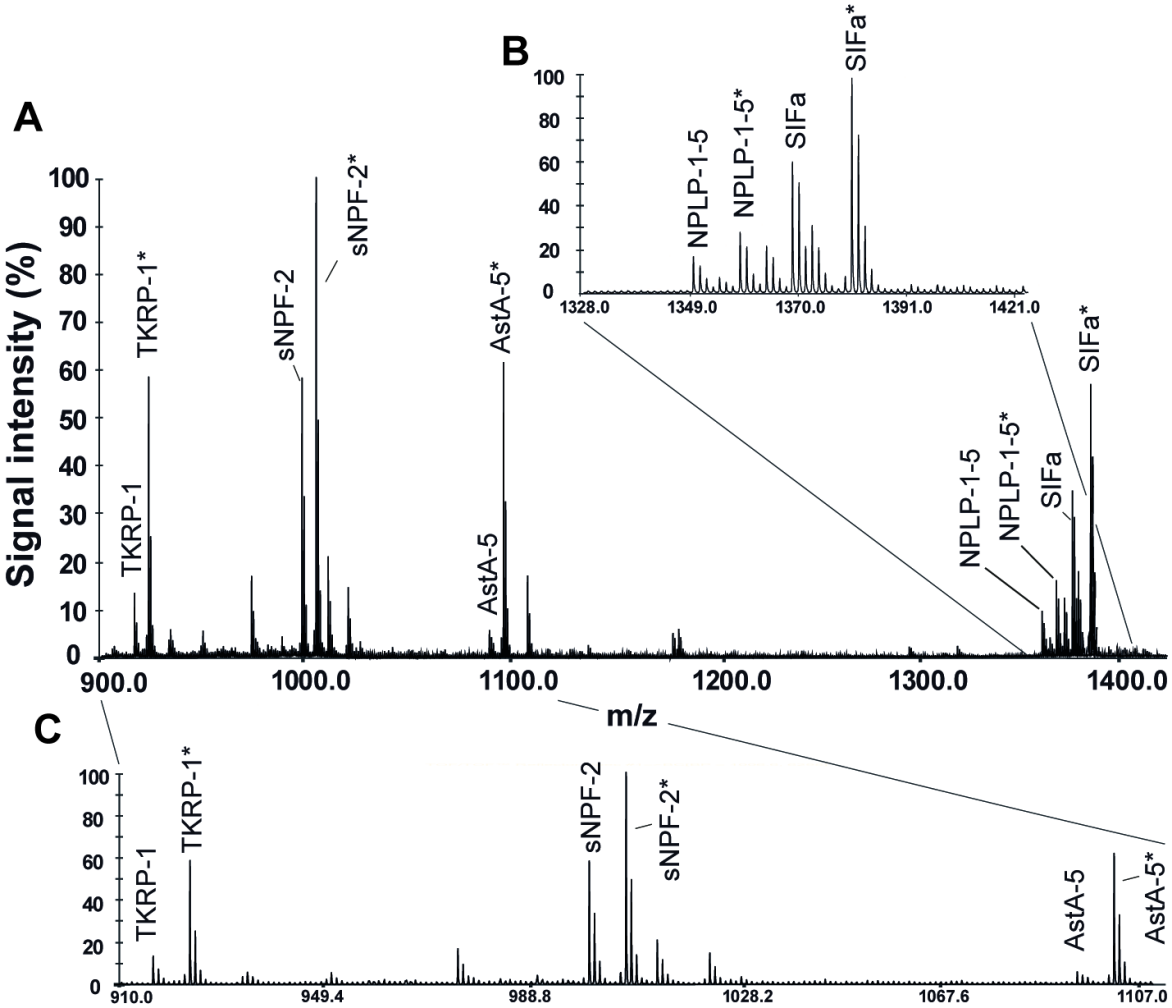
Representative MALDI-TOF MS spectrum of a single antennal lobe from a female *Aedes aegypti* 48 h post-blood meal. **A**. Signal intensity of tachykinin-related-peptide-1 (TKRP-1), short neuropeptide F-2 (sNPF-2), allatostatin-A-5 (AstA-5), neuropeptide-like-precursor-1-5 (NPLP-1-5), and SIFamide (SIFa), along with their isotope labelled standards (indicated by *). **B-C**. Magnification of A. Left y-axis: relative signal intensity after autoscaling to maximum peak intensity in the selected mass range.

Data inspection, baseline correction and peak deisotoping was performed, using the software Data Explorer (version 4.10; Applied Biosystems/MDS Analytical Technologies Instruments, Framingham, USA). Spectra were discarded when a peak representing one of the five investigated neuropeptides was below detection level (less than 5% above baseline), or in the event of an unusual pattern of peaks appearing, according to Siju et al. (2014). Peak lists were exported from Data Explorer into Microsoft Excel (Microsoft Office 2007; Microsoft, CA, USA). Peaks of the neuropeptides and the internal standards (Fig 1) were identified by comparing the m/z with the calculated masses (Table 1). The five neuropeptide peaks were mass-identical with the corresponding neuropeptides previously identified by tandem mass spectrometry [37]. Signal intensities of intrinsic peptides were divided by the signal intensities of the synthetic peptides, resulting in the signal intensity ratio (sir) as a measure for the neuropeptide amount in the tissue. The sir for individual mosquitoes was calculated by using the mean sir of both ALs, if present.

For statistical analysis, the samples were grouped according to the feeding state of the animals into nbf, bf, and blood- and sugar-fed females (bf+s). A general linear model was used, taking the methodological replicates (day effect) into account as a second factor. Data were tested for normal distribution using a Kolmogorov-Smirnov test, and in the event that normality could not be assumed, the data were transformed using the natural logarithm. *Post-hoc* analysis was performed using Fisher’s exact test. Statistical analysis was carried out in Minitab 16 (Minitab Statistical Software, State College, PA, RRID: SCR_014483). Data were plotted using Graph Pad Prism 6.

### Neuropeptide injection

Female *A*. *aegypti* were cold anesthetized and injected using a Nanoliter Injector 2000 (World Precision Instruments, Sarasota, FL, USA). For each mosquito, 100 nL physiological saline [38], alone or in combination with 10^−2^ M or 10^−3^ M of synthetic neuropeptide (sNPF-2, AstA-5, NPLP-1-5 or SIFamide; Peptides & Elephants GmbH, Potsdam, Germany), was injected into the thorax, aiming between the post-spiricular and the pre-alar areas, using glass capillary needles made using a PP-830 Glass Microelectrode Puller (Narishige, London, U.K). Additionally, a blend of sNPF (10^−2^ M) and AstA (10^−2^ M) was tested. For technical reasons no blends involving NPLP-1-5 were possible, due to lack of solubility of this neuropeptide in the stock solution required for obtaining the 10^−2^ M blend. SIFamide was included in the test panel as a negative control. Injected mosquitoes were allowed to rest for 1 h, and then tested in groups of 30–40 in the wind tunnel assay, described above. The number of mosquitoes attracted to the odor source was determined five minutes after opening the release cage. For each experiment, six biological replicates were performed and the mean percentage of the mosquitoes responding was determined. The behavioral response of neuropeptide and saline injected animals were compared using a one way ANOVA followed by a Tukey’s honestly significant difference (HSD) *post-hoc* test (Graph Pad Prism 6). As we found no significant differences among our controls, these were pooled and used in subsequent Tukey’s (HSD) *post-hoc* analyses.

## Results

### Blood feeding suppresses host-seeking behavior

**Fig 2:**
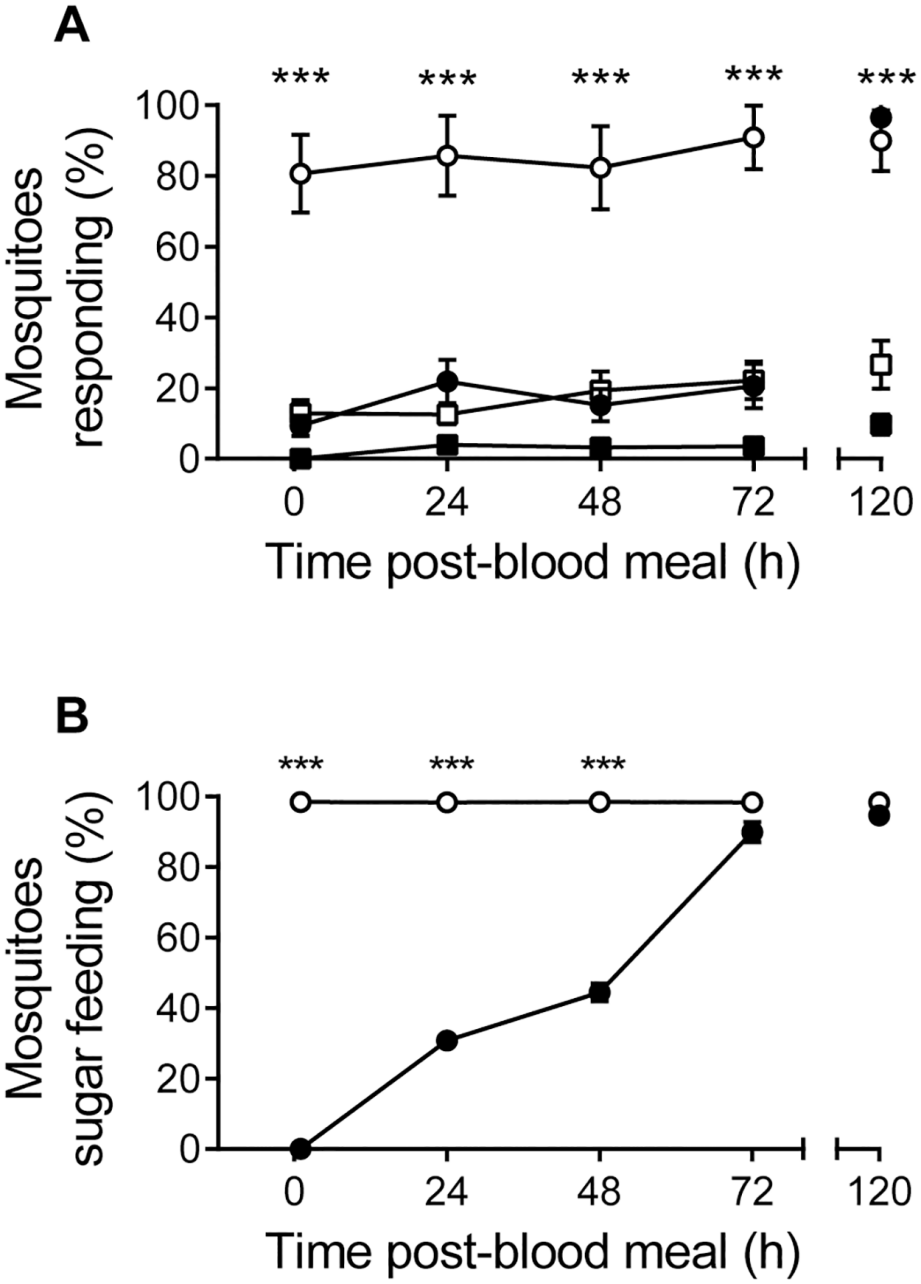
Host-seeking and sugar feeding in female *Aedes aegypti* after a blood meal. **A**. Percentage of non-blood fed (white) and blood fed (black) female *A*. *aegypti* (n ≈ 30) responding to odors emanating from a human hand (circle) or to a gloved hand as a control (square). Mosquitoes were allowed to lay eggs 96 h post-blood meal. Level of significance for the nominal logistic regression model is shown. **B**. Percentage of non-blood fed (white) and blood fed (black) female *A*. *aegypti* (n ≈ 30–50) taking a sugar meal following a blood meal. Mosquitoes were allowed to lay eggs 96 h post-blood meal. Significant differences between groups were tested using Fisher’s exact test. ***, *p* < 0.001.

Blood-feeding significantly inhibited the host-seeking behavior of female *A*. *aegypti* immediately following a complete blood meal, an effect lasting until after egg-laying (Fig 2A). This effect was independent of sugar feeding (1 h, *p* > 0.999; 24 h, *p* > 0.999; 48 h pbm, *p* > 0.999; 72 h pbm, *p* = 0.206; 120 h pbm, *p* > 0.999). The behavioral response to human odor by blood-fed mosquitoes was significantly reduced compared to non-blood fed mosquitoes, when tested at 1 h, 24 h, 48 h and 72 h pbm (*p* < 0.0001). From 1 h, to 72 h pbm, no significant differences were observed in the response of the bf and nbf mosquitoes in the non-odor control (1 h, *p* = 0.656; 24 h, *p* = 0.656; 48 h, *p* = 0.656; 72 h, *p* = 0.877). The behavioral inhibition to host odors recovered fully 24 h post-egg laying (120 h pbm, *p* = 0.306).

### Sugar feeding is transiently suppressed after blood feeding

A complete blood meal transiently suppressed sugar meal consumption (Fig 2B). Non-blood fed mosquitoes, when offered a sugar meal for 1 h per day, consistently imbibed a sugar meal (Fig 2B). In contrast, when tested 1 h after a blood meal, females did not imbibe a sugar meal, an inhibition that was gradually released over the course of the next three days, and culminated in a complete restoration with the onset of pre-oviposition behavior (1 h pbm, *p* < 0.0001; 24 h pbm, *p* < 0.0001; 48 h pbm, *p* < 0.0001; 72 h pbm, *p* = 0.068; 120 h pbm, *p* = 0.426).

### Blood feeding and sugar feeding influence neuropeptide levels

**Fig 3:**
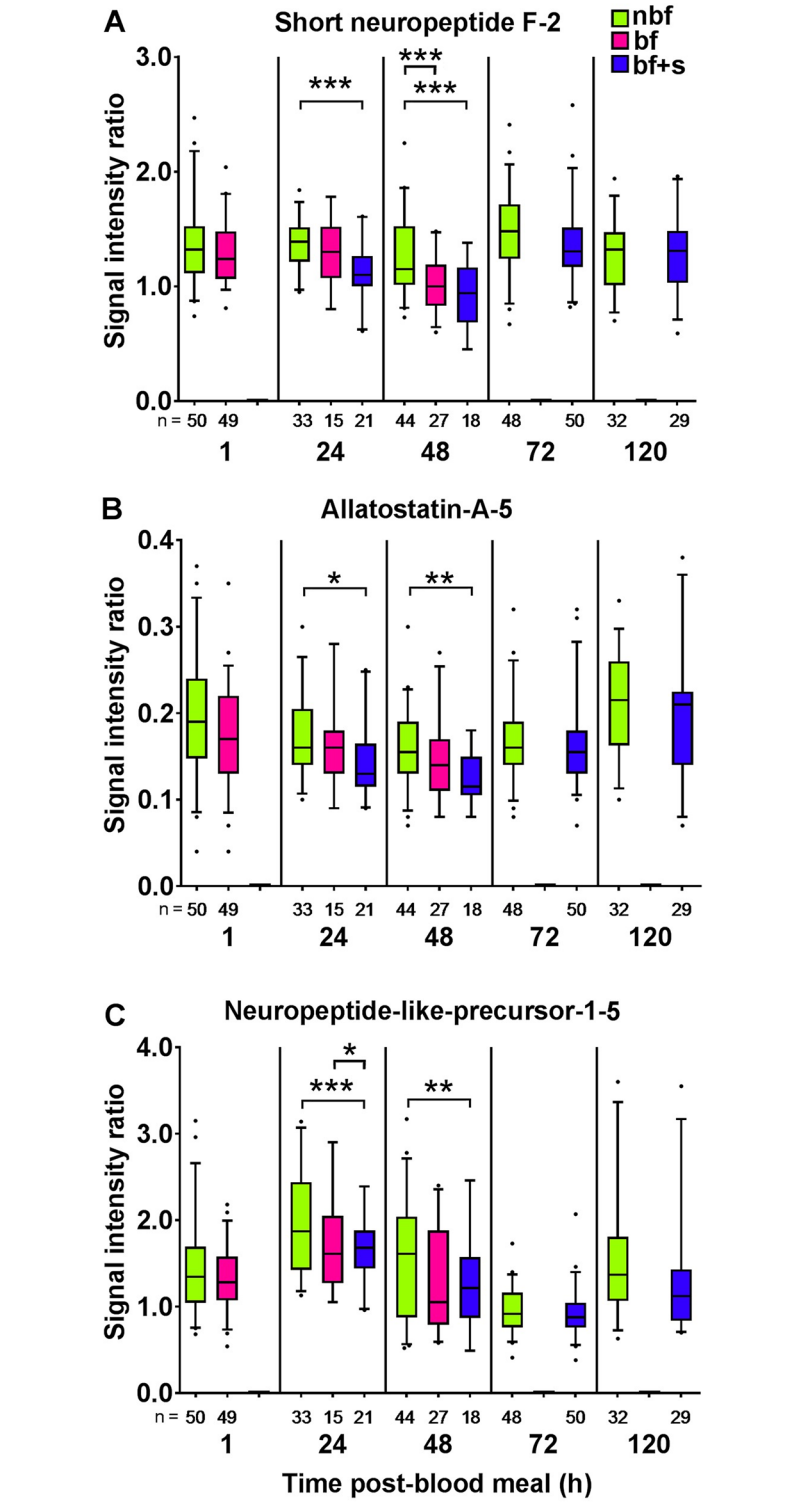
Regulation of neuropeptide levels in the antennal lobes of *Aedes aegypti* females in response to different feeding regimes. Box plots with whiskers representing the 5–95% percentile of signal intensity ratios of short neuropeptide F-2 (**A**), allatostatin-A-5 (**B**), and neuropeptide-like-precursor-1-5 (**C**) at 1 h, 24 h, 48 h, 72 h, and 120 h post-blood meal (pbm). The bar represents the 25–75 percentile, the line the median and the dots data points outside the 5–95% range. Nutritional state conditions of the mosquitoes are non-blood fed but sugar fed (nbf), blood fed (bf), and blood and sugar fed (bf+s). Due to transient sugar feeding inhibition of bf mosquitoes no data are available for bf+s females 1 h pbm. As mosquitoes consistently consumed sugar throughout the rest of the experiment, there are no data for bf females at 72 h and 120 h pbm. The n-values indicate the number of biological replicates of ALs from individual mosquitoes. Data were analyzed using a general linear model for each respective time point. Asterisks indicate significant differences between groups. Significance levels: *, *p* < 0.05; **, *p* < 0.01; ***, *p* < 0.001.

For the MALDI-TOF MS analyses, we investigated the levels of selected AL neuropeptides in non-blood fed but sugar fed (nbf; recently fed), blood fed (bf; ≥24 h unfed), and blood and sugar fed (bf+s; recently fed) mosquitoes to assess the effect of feeding regime on neuropeptide expression. The rationale for including three different feeding regimes was to investigate the effect of both feeding (recently fed vs ≥24 h unfed) and diet (sugar, blood or mixed) on the neuropeptide levels. The observed behavioral inhibition after a blood meal or a blood and sugar meal corresponded to a significant, and selective, decrease in sNPF-2, AstA-5 and NPLP-1-5 levels in the ALs (Fig 3), but not in SIFa and TKRP-1 (*p* > 0.05). Compared to that of nbf female ALs, the combination of a blood meal followed by a sugar meal resulted in a reduction in the level of sNPF-2, AstA-5 and NPLP-1-5 in the ALs, 24 h pbm (sNPF-2, *p* = 0.001; AstA-5, *p* = 0.037; NPLP-1-5, *p* < 0.001) and 48 h pbm (sNPF-2, *p* < 0.001; AstA-5, *p* = 0.004; NPLP-1-5, *p* = 0.003). A blood meal alone did not elicit a change in neuropeptide levels that differed from nbf females, except in the case of sNPF-2 at 48 h pbm (*p* < 0.001). Additionally, there was no significant difference in levels of neuropeptides between bf and bf+s ALs, except for NPLP-1-5 at 24 h pbm (*p* = 0.017). In conclusion, modulation of neuropeptide levels in the ALs is influenced differentially and is dependent on the feeding regime.

### Injection of sNPF-2 and AstA-5 inhibits host seeking behavior

The MALDI-TOF MS analyses indicated that sNPF-2, AstA-5 and NPLP-1-5 may play a role in regulating olfactory processing in the AL, and thereby affecting the odor-mediated host seeking of female *A*. *aegypti*. To assess the functional significance of sNPF-2, AstA-5 and NPLP-1-5, these neuropeptides were systemically injected into nbf animals. In addition, SIFa as a negative control. A significant reduction in host seeking behavior was observed following injection with 10 mM of either sNPF-2 (*p* < 0.001) or AstA-5 (*p* < 0.001), but not with 1 mM (*p* > 0.05). Systemic injection of NPLP-1-5 (10 mM), however, had no effect (*p* = 0.815). As a control, we injected SIFa, which was not regulated according to our mass spectrometric analysis, and observed no changes in host seeking behavior (*p* > 0.109).

**Fig 4:**
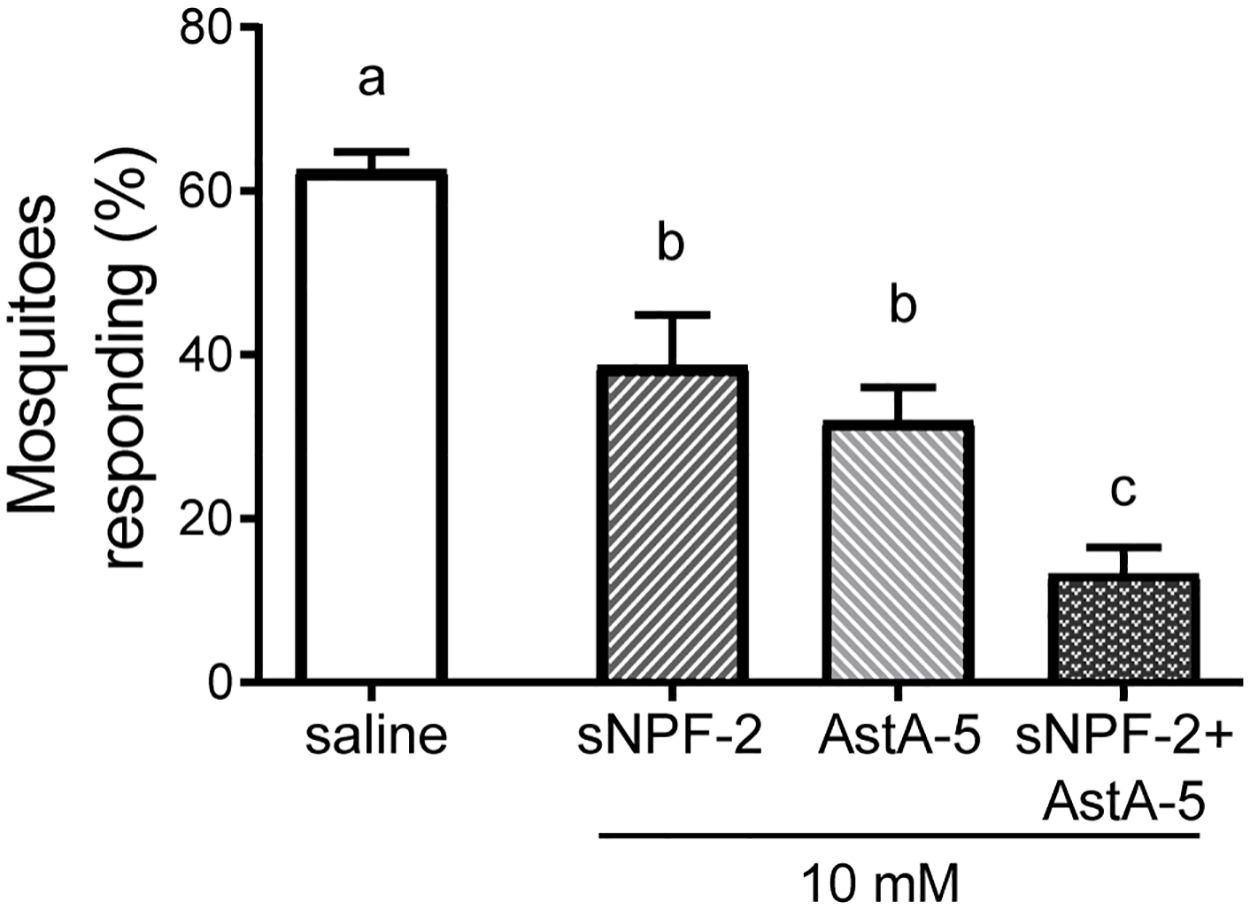
Systemic injection of synthetic short neuropeptide F-2 (sNPF-2) and allatostatin-A-5 (AstA-5) into non-blood fed female *A*. *aegypti* inhibits host seeking behavior. The percentage of mosquitoes responding to human host cues after injection of physiological saline or 10 mM of sNPF-2, AstA-5, or a blend of both neuropeptides, is presented. As the behavior of physiological saline injected animals did not differ among the replicates, the data were pooled for comparison. Six biological replicates were performed, and for each group 30–40 mosquitoes were tested. All data are plotted as mean ± SEM. Bars that are not significantly different share the same letter.

Injection of either 10 mM of sNPF-2 or AstA-5 alone was not sufficient to abolish host seeking behavior completely, therefore we injected a binary mixture of the two neuropeptides (10 mM) and found a significant reduction in the response to host cues compared to sNPF-2 (*p* = 0.001) and AstA-5 (*p* = 0.036; Fig 4). This reduction in response to host cues was similar to that observed in bf females (Fig 2).

## Discussion

Blood feeding in *A*. *aegypti* induces a transient inhibition of both sugar feeding and host seeking. Here, we demonstrate that neuropeptides both regulate and are regulated by feeding-related behaviors. Using MALDI-TOF MS, we show that the transient inhibition of both sugar feeding and host seeking correlates with significant, transient changes in the neuropeptide levels of sNPF-2, AstA-5, and NPLP-1-5 in the ALs following a blood meal, and is dependent on the feeding regime. Systemic injection of sNPF-2 and AstA-5 in nbf females suggest that a dual neuromodulatory pathway regulates the odor-mediated host seeking behavior in *A*. *aegypti* females.

### Blood feeding transiently suppresses sugar feeding and host seeking

Host seeking behavior of female *A*. *aegypti* is significantly inhibited following a complete blood meal. These results are in line with those previously shown for both *A*. *aegypti* and *Anopheles gambiae* [4,6,19,39]. In addition, a complete blood meal transiently suppressed sugar meal consumption, reflecting the mutual inhibition of feeding responses during consumption and digestion [40]. The initial period of the post-blood meal inhibition of sugar seeking and feeding is likely caused by abdominal distension, which also inhibits host seeking [18]. Although not tested here, previous studies have shown that the behavioral response to flower odors is inhibited following a blood meal, persisting until approximately 72 h pbm [41]. These findings are in accordance with observations of wild mosquitoes, in which females fully engorged with blood are rarely found nectar feeding [42]. In contrast gravid mosquitoes, approximately 72 h pbm, frequently replenish their energy resources before oviposition, which can take the form of either a blood or sugar meal [23–25]. Under laboratory conditions, this supplementary meal is usually provided as a sugar meal [4,6]. In the absence of a sugar meal, the responsiveness to host cues returns fully between 48 h and 72 h pbm [24]. It is interesting to note that the time course of behavioral inhibition following a meal differs according to the meal type imbibed, sugar or blood, suggesting that these processes are, at least in part, differentially regulated. The significance of the restoration of sugar feeding post-blood meal is for females to replenish their energy resources to extend life expectancy [22,43,44]. Sugar feeding also affects reproductive success by increasing follicle development before a blood meal during the previtellogenic phase (up to 72 h post-emergence), and by providing sufficient energy so that subsequent blood meals can be used directly in vitellogenin production and deposition [22,23,40,45].

### Blood and sugar feeding influence neuropeptide levels

The observed behavioral inhibition after a blood meal corresponded to a significant, and selective, decrease in sNPF-2, AstA-5, and NPLP-1-5 levels in the ALs, between 24 and 48 h pbm that was dependent on the diet of the female. Both nbf and bf+s females consumed a sugar meal, and by including both of these feeding regimes in the MALDI-TOF analysis, we are able to describe the changes in AL neuropeptide levels as a response to blood feeding. From this we were able to tentatively identify the neuropeptides involved in regulating the odor-mediated host seeking behavior. Besides the effect observed in response to blood feeding, changes in the levels of sNPF-2 and NPLP-1-5 in the ALs were also observed in response to sugar feeding (comparing nfb/bf+s with bf females). Thus, sugar and blood meals differentially affect AL neuropeptide levels.

Short neuropeptide F and AstA have previously been associated with the regulation of feeding-related behaviors in various insect species, including *A*. *aegypti* [6–8,10,46–49]. When injected at high concentrations into the abdomen, sNPF-3 and the structurally-related Head Peptide-I partially inhibit host seeking in non-blood fed *A*. *aegypti* with *ad libitum* access to sugar [5,6]. Our results reveal that sNPF-2 has a similar effect on host seeking as sNPF-3 in *A*. *aegypti* [6]. The combination of the semi-quantitative analysis with the functional analyses suggests that the observed changes are due to a functional consumption of sNPF-2 and AstA-5 through release and depletion of the stores from AL neurons, resulting in a reduction in host seeking in blood fed females. These results are in line with observations made in *B*. *mori*, which demonstrate that the functional consumption of sNPF in starved animals correlates with a change in feeding activity [48,50].

Studies in other insects support negative [6,47,51–53] as well as positive effects of sNPF on feeding behavior [49,50,54,55]. In *D*. *melanogaster*, the neural mechanism underlying this behavior has been studied in some detail. In this species, sNPF is expressed in a subset of OSNs and facilitates odor-mediated feeding behavior [31,56]. In addition, sNPF regulates starvation-induced attraction towards food odors by presynaptic facilitation of OSNs [7,10]. Ko et al. (2015) proposed that sNPF sensitizes the olfactory system to attractive food-related odors by upregulating the expression of the sNPF receptor in starved flies, while sNPF is released independent of the feeding status. In contrast, systemic injection of sNPF decreases food uptake in the locust *Schistocerca gregaria*, while systemic knock-down by RNAi has the opposite effect [52,53]. Moreover, in the red fire ant, starvation leads to a decrease of the sNPF receptor expression [51]. The mechanism by which sNPF acts on the CNS in these insects, as well as in the ALs of *A*. *aegypti*, to elicit changes in feeding behavior, merits further investigation.

The observed change in AstA-5 AL levels was dependent on the combined ingestion of blood and sugar by female *A*. *aegypti*. Moreover, injection of AstA-5 reduced host seeking in nbf females, revealing a functional role of AstA in its regulation. Modulation of the AstA system has previously been shown for *R*. *prolixus*, in which the changes in responsiveness to host odors [57] coincide with a decrease in AstA transcripts in the central nervous system post-blood meal [58]. In *D*. *melanogaster*, AstA inhibits feeding behavior [8] and guides the decision to feed on either protein or sugar, depending on metabolic needs [11]. So far, nothing is known about the direct effects of AstA on the olfactory system in insects. Similar to *D*. *melanogaster*, AstA is expressed in AL LNs of *A*. *aegypti* [32]. These neurons act through lateral excitation or inhibition to shape the responses of OSNs and projection neurons [59]. Investigations into the mechanism by which AstA acts on the ALs of *A*. *aegypti* to elicit changes in feeding behavior may lead to a better understanding of the regulation of host seeking in this disease vector.

Interestingly, the injection of a binary mixture of sNPF and AstA lead to a reduction of host seeking, at a level similar to that observed in blood fed mosquitoes. This strongly suggests that neuropeptides released in concert and acting on different receptors are required for the regulation of host seeking, as also concluded by Liesch et al. (2013). Similarly, Nagata et al. (2012) identified changes in both sNPF and AstA in their semi-quantitative mass spectrometric analysis of *B*. *mori* larva brains, indicating a complex modulation of these neuropeptide signaling pathways in response to feeding.

In contrast to the injection of sNPF-2 and AstA-5, that of NPLP-1-5 did not lead to a change in host seeking. Changes in NPLP-1 levels have previously been reported in *R*. *prolixus* in response to blood feeding [9]. These authors showed a differential regulation of two NPLP-1 peptides following a blood meal, with one increasing 4 h pbm and then, together with the second, decreasing 24 h pbm compared with nbf individuals. So far, the function of NPLPs in insects remains elusive.

## Conclusion

This study shows that the selective and differential modulation of specific AL neuropeptides in *A*. *aegypti* is dependent on feeding regime. The functional characterization provides strong evidence of a dual neuromodulatory system involved in the regulation of olfactory processing in the primary olfactory center following a sugar and/or blood meal. The increasing availability of gene editing tools for insects other than *D*. *melanogaster* [60], along with advances in imaging techniques [7,10,61], will allow for a more in depth analysis of the neuropeptidergic modulation of feeding behaviors in *A*. *aegypti* in the future.

## Supporting information

S1 File Minimal data set for Figs 2, 3 and 4. (XLSX)
